# Host gene response to endosymbiont and pathogen in the cereal weevil *Sitophilus oryzae*

**DOI:** 10.1186/1471-2180-12-S1-S14

**Published:** 2012-01-18

**Authors:** Aurélien Vigneron, Delphine Charif, Carole Vincent-Monégat, Agnès Vallier, Frédérick Gavory, Patrick Wincker, Abdelaziz Heddi

**Affiliations:** 1 INSA-Lyon, UMR203 BF2I, INRA, Biologie Fonctionnelle Insectes et Interactions, Bat. Louis-Pasteur 20 ave. Albert Einstein, F-69621 Villeurbanne, France; 2 UMR CNRS 5558 Laboratoire de Biométrie et Biologie Evolutive, Université Claude Bernard Lyon, F-69621 Villeurbanne, France; 3 Commissariat à l'Énergie Atomique (CEA), Genoscope (Centre National de Séquençage), 2 rue Gaston Crémieux, CP 5706, 91,057 Evry Cedex, France

## Abstract

**Background:**

Insects thriving on nutritionally poor habitats have integrated mutualistic intracellular symbiotic bacteria (endosymbionts) in a bacteria-bearing tissue (the bacteriome) that isolates the endosymbionts and protects them against a host systemic immune response. Whilst the metabolic and physiological features of long-term insect associations have been investigated in detail over the past decades, cellular and immune regulations that determine the host response to endosymbionts and pathogens have attracted interest more recently.

**Results:**

To investigate bacteriome cellular specificities and weevil immune responses to bacteria, we have constructed and sequenced 7 cDNA libraries from *Sitophilus oryzae* whole larvae and bacteriomes. Bioinformatic analysis of 26,886 ESTs led to the generation of 8,941 weevil unigenes. Based on *in silico* analysis and on the examination of genes involved in the cellular pathways of potential interest to intracellular symbiosis (*i.e.* cell growth and apoptosis, autophagy, immunity), we have selected and analyzed 29 genes using qRT-PCR, taking into consideration bacteriome specificity and symbiosis impact on the host response to pathogens. We show that the bacteriome tissue accumulates transcripts from genes involved in cellular development and survival, such as the apoptotic inhibitors *iap2* and *iap3*, and endosomal fusion and trafficking, such as *Rab7*, *Hrs*, and *SNARE*. As regards our investigation into immunity, we first strengthen the bacteriome immunomodulation previously reported in *S. zeamais.* We show that the sarcotoxin, the c-type lysozyme, and the *wpgrp2* genes are downregulated in the *S. oryzae* bacteriome, when compared to aposymbiotic insects and insects challenged with *E. coli*. Secondly, transcript level comparison between symbiotic and aposymbiotic larvae provides evidence that the immune systemic response to pathogens is decreased in symbiotic insects, as shown by the relatively high expression of *wpgrp2*, *wpgrp3*, coleoptericin-B, diptericin, and sarcotoxin genes in aposymbiotic insects.

**Conclusions:**

Library sequencing significantly increased the number of unigenes, allowing for improved functional and genetic investigations in the cereal weevil *S. oryzae*. Transcriptomic analyses support selective and local immune gene expression in the bacteriome tissue and uncover cellular pathways that are of potential interest to bacteriocyte survival and homeostasis. Bacterial challenge experiments have revealed that the systemic immune response would be less induced in a symbiotic insect, thus highlighting new perspectives on host immunity in long-term invertebrate co-evolutionary associations.

## Background

Bacterial intracellular symbiosis (endosymbiosis) is widespread in invertebrates and exhibits a large variety of phenotypes, ranging from mutualism to pathogenesis. Endosymbionts are transmitted vertically for hundreds of host generations and affect the host biology in many ways, including reproduction, physiology and behavior [1-4]. The outcome of the association depends on the interactional networks between the host and bacterial partners, which sometimes interfere concomitantly with many cellular features such as metabolism, apoptosis and immunity [5-7].

Insects living on unbalanced nutritional diets house so-called obligate endosymbionts, which interfere in the early stages of host embryogenesis with the differentiation of specialized host cells (the bacteriocytes) that isolate the endosymbionts and protect them from the host immune systemic response [6,8]. In addition to the primary endosymbiont, which is fixed in all host populations and is essential for host fitness and survival, insects may integrate, during their evolutionary history, secondary endosymbionts that are facultative and have an impact on other biological and ecological features of the host [9,10]. Evidence of symbiont elimination and displacement has also been reported in weevils [11,12] and suspected in other insect groups where multiple bacterial species are coexisting within a single host lineage [13,14].

Once established within the host, endosymbionts can experience severe genome size reduction due to relaxed evolutionary pressures on the genes that are unnecessary or redundant with respect to the host functions [15-17]. As reported in *Sodalis*, the secondary endosymbiont of the tsetse fly, gene mutation and deletion processes can also affect cell membrane components and genes encoding Microbe-Associated Molecular Patterns (MAMPs) [18]. As these elements are essential for bacterial perception by the host immune system, the complexity of molecular cross-talk between partners may evolve according to the level of bacterial genomic degeneration and, hence, according to the age of the association. However, while physiological and evolutionary aspects of insect endosymbiosis have been thoroughly investigated over the past decades, very little is known about the molecular mechanisms that permit the establishment of symbiosis and then the maintenance and the regulation of symbiotic intracellular bacteria. Important questions concern, first, how endosymbionts are recognized and tolerated by the host immune system, secondly how cellular pathways are regulated to prevent bacteriocyte cell disorders and death due to chronic infection with endosymbionts and, thirdly, how does endosymbiosis influence host immunocompetence directed at pathogens?

In *Drosophila melanogaster*, microbe recognition leads to signal production via four pathways (Toll, Immune Deficiency (IMD), JNK, and JAK/STAT) [19-21]. Each pathway responds to particular types of pathogens, i.e. Gram-positive bacteria and fungi for Toll and Gram-negative bacteria for IMD. Signalling through the Toll receptor activates a set of phosphorylating reactions involving complex adaptors. An inhibitor protein, called Cactus, is degraded, thus releasing its associated nuclear factor protein, called Dorsal-related Immunity Factor (DIF), which translocates into the nucleus and induces antimicrobial peptide genes. The Imd protein is upstream of two separate pathways. The first pathway involves a protein from the mitogen-activated protein (MAP) 3 kinase family, the dTAK1 (*Drosophila* transforming growth factor β activated kinase 1) associated with dTAB2 (*Drosophila* TAK1 Binding) [22] and requiring the potential E3 ubiquitin ligase dIAP2 (*Drosophila* Inhibitor of APoptosis2) [23-25]. The latter appears to be a good candidate for activating the IKK (inhibitor kB kinase) signalosome proteins, which in turn phosphorylate the Relish (Rel family) transcriptional factor. The second pathway controls the cleavage of Relish. The “*Drosophila* Fas-associated death-domain-containing protein” (dFADD), which is homologous to the mammalian adaptor protein that interacts with the complex “tumor necrosis factor receptor 1” (TNF-R1) to recruit pro-caspase-8, links IMD to the caspase “death-related ced-3/Nedd2-like” (DREDD) in order to build the “adaptor” complex that allows the activation of caspases and apoptosis [26,27]. This pathway may end with a proteasome-independent proteolytic cleavage of Relish, probably by the DREDD protein [28,29]. The Relish cleavage dissociates the Rel and the Ankyrins and allows for processing of the nuclear transcriptional factor.

To investigate immune and cellular processes in the bacteriome tissue, we have used cereal weevils as a symbiotic system [6,30]. These crop pests include three species (*i.e. Sitophilus oryzae*, *Sitophilus zeamais and Sitophilus granarius*) that all have in common an intracellular symbiosis with a Gram-negative γ-Proteobacterium, called *Sitophilus* primary endosymbiont (or SPE) [31,32]. *Sitophilus* insects provide an interesting system for studying host immune responses to symbionts as their association with SPE was established relatively recently (less than 25 MY ago), probably by endosymbiont replacement [11,12,17]. The endosymbiont genome has not experienced severe gene deletion [17,33]. It encodes functional secretion systems [34] and genes encoding cell wall elements (unpublished data). Using suppressive subtractive hybridization (SSH), we have already identified several immune-relevant genes of *S. zeamais* species and we have demonstrated that weevil bacteriomes exhibit a specific local immune expression that allows symbiont persistence within the bacteriocyte cells [6].

Here, we have studied the sibling *S. oryzae* species. We have enlarged the panel of genes potentially involved in host-symbiont interaction through the construction and the sequencing of 7 different libraries from whole larvae and from bacteriomes (i.e. SSH, non-normalized and normalized libraries). Bioinformatic analysis of 26,886 ESTs has generated 8,941 unigenes. The results of qRT-PCR experiments strongly support the gene expression profile previously reported for the *S. zeamais* bacteriome [6], uncover new genes involved in the immune system, apoptosis, vesicular trafficking and cell-growth in the bacteriome tissue, and broaden the proposal that endosymbiosis may influence the host immune response in long-term host-symbiont coevolution.

## Methods

This work has been conducted in parallel with two other invertebrate models (i.e. *Armadillidium vulgare/Wolbachia* and *Asobara tabida*/*Wolbachia*) with the object of identifying conserved and divergent immune pathways and to determine whether invertebrates have selected common strategies to control their symbionts and to discriminate between symbionts and pathogens [35,36].

### Insect manipulation and sample preparation

Insects used in this study were reared on wheat grains at 27.5°C and at 70% relative humidity (rh). *Sitophilus* weevils house both the integrated endosymbiont SPE and the facultative endosymbiont *Wolbachia *[3]. To avoid any side effects from *Wolbachia*, the “Bouriz” *S. oryzae* strain was chosen because it harbors SPE only. SPE-free aposymbiotic insects were obtained as described previously [37].

Bacteriomes were dissected from fourth instar larvae in Buffer A (25nM KCl, 10nM MgCl2, 250nM Sucrose, 35nM Tris/HCl, pH=7.5), and stored at -80°C prior to RNA preparation.

To identify genes involved in the immune response, we challenged fourth instar larvae with the intracellular bacteria *Salmonella typhimurium* (*Salmonella*, Strain 12023G). About 10^5^ bacteria were injected into the weevil hemolymph, using a Nanoject II apparatus (Drummond, Broomall, PA). The larvae were incubated for 3, 6 or 12 hours at 27.5°C and 70% rh and then stored at -80°C until required for RNA preparation.

### Library constructions

Details of material and conditions used for library constructions are summarized in Table 1.

**Table 1:** Libraries description and construction method.

	*Library*	*Type*	*Origin*	*Status of infection*	*Presence of symbiont*	*Description*	*Number of individuals / bacteriomes sampled and pooled* (*quantity of RNA used from samples*)
Host response to pathogen	SSH1	Subtraction	Whole larvae	infected	no	*Salmonella*+ *vs*. *Salmonella*-	*Salmonella* -: 10 uninfected aposymbiotic larvae (10µg)
	SSH2	Subtraction	Whole larvae	Not infected	no	*Salmonella*- *vs. Salmonella*+	*Salmonella* +: 15 infected aposymbiotic larvae: 5 collected 3h after infection (3.33µg), 5 after 6h (3.33µg) and 5 after 12h (3.33µg)

Host response to symbiont	SSHA	Subtraction	Bacteriome	Not infected	yes	With symbiont *vs.* without symbiont	With symbiont: 200 symbiotic bacteriomes (10 µg)
	SSHB	Subtraction	Bacteriome	Not infected	no	Without symbiont *vs.* with symbiont	Without symbiont: 640 aposymbiotic bacteriomes (10 µg)
	
	SO	Non-normalized	Bacteriome	Not infected	yes	Pool of bacteriomes with symbiont	170 symbiotic bacteriomes (10µg)
	
	AO	Non-normalized	Bacteriome	Not infected	no	Pool of bacteriomes without symbiont	578 aposymbiotic bacteriomes (10 µg)

	NOR	Normalized	Whole larvae	infected	yes	Pool of Symbiotic Larvae / Aposymbiotic larvae / Aposymbiotic larvae infected during 3h, 6h and 12h	10 uninfected aposymbiotic larvae (2µg) / 10 uninfected symbiotic larvae (2µg) / 15 infected aposymbiotic larvae: 5 collected after 3h of infection (2µg), 5 after 6h (2µg) and 5 after 12h (2µg)

Total RNA was extracted with TRIzol Reagent (Invitrogen, Cergy-pontoise, France), following the manufacturer’s instructions. RNA was purified using the RNeasy mini kit (QIAGEN, Alameda, CA) following the “RNA Clean Up” protocol. After purification, the RNA concentration of each sample was measured with a Nanodrop® spectrophotometer (Thermo Scientific, Wilmington, DE) and total RNA quality was checked by electrophoresis.

#### Libraries prepared from bacteriome tissue

SO (symbiont-full bacteriome) and AO (symbiont-free bacteriome) Libraries (see Table 1) were prepared using the Creator SMART cDNA Library Construction kit (Clontech/BD Biosciences, PaloAlto, CA), following the manufacturer’s instructions. cDNA was digested with Sfi1, purified (BD Chroma Spin – 400 column) and then ligated into a pDNRlib vector for *E. coli* transformation.

#### SSH

SSHA (symbiont-full/symbiont-free bacteriome), SSHB (symbiont-free/symbiont-full bacteriome), SSH1 (Challenged/Non-Challenged with *S. typhimurium*) and SSH2 (Non-Challenged/Challenged with *S. typhimurium*) were performed by Evrogen (Moscow, Russia). In order to reduce the number of false-positive clones in the SSH-generated libraries, the SSH technology was combined with a mirror orientation selection procedure [38]. Purified cDNA were cloned into the pAL16 vector (Evrogen, Moscow, Russia) and used for *E. coli* transformation.

#### Normalized library

NOR was prepared by Evrogen (Moscow, Russia). Total RNA was used for ds cDNA synthesis using the SMART approach [39]. SMART prepared amplified cDNA was then normalized according to [40]. Normalization included cDNA denaturation and reassociation, using treatment with duplex specific nuclease (DSN), as described by [41]. Normalized cDNA was purified using a QIAquick PCR Purification Kit (QIAGEN, Alameda, CA), digested with restriction enzyme Sfi1, purified (BD Chroma Spin - 1000 column), and ligated into a pAL 17.3 vector (Evrogen, Moscow, Russia) for *E. coli* transformation.

### EST sequencing and data processing

**Figure 1:**
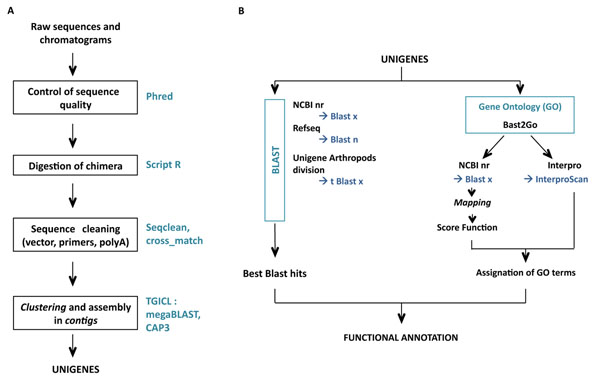
Sequence treatment (A) and functional annotation procedure (B).

All clones from the libraries were sequenced using the Sanger method (Genoscope, Evry, France) and were deposited in the GenBank database. A general overview of the EST sequence data processing is given in Figure 1. Raw sequences and trace files were processed with Phred software [42,43] in order to remove any low quality sequences (score < 20). Sequence trimming, which includes polyA tails/vector/adapter removal, was performed by cross_match. Chimerical sequences were computationally digested into independent ESTs.

Clustering and assembly of the ESTs were performed with TGICL [44] to obtain unique transcripts (unigenes) composed of contiguous ESTs (contigs) and unique ESTs (singletons). For this purpose, a pairwise comparison was first performed using a modified version of megablast (minimum similarity 94%). Clustering was performed with tclust, that works via a transitive approach (minimum overlap: 60bp to 20bp maximum from the end of the sequence). Assembling was carried out with CAP3 (minimum similarity 94%).

To detect unigene similarities with other species, several blasts (with high cut-off e-values) were performed against the following databases: NCBI nr (blastx (release: 1 March 2011); e-value < 5, HSP length > 33aa), Refseq genomic database (blastn, e-value < 10), Unigene division Arthropods (tblastx, #8 *Aedes aegypti*, #37 *Anopheles gambiae*, #3 *Apis mellifera*, #3 *Bombyx mori*, #53 *Drosophila melanogaster*, #9 *Tribolium castaneum*; e-value < 5). Gene Ontology annotation was carried out using blast2go software [45]. In the first step (mapping), a pool of candidate GO terms was obtained for each unigene by retrieving GO terms associated with the hits obtained after a blastx search against NCBI nr. In the second step (annotation), reliable GO terms were selected from the pool of candidate GO terms by applying the Score Function (FS) of Blast2go with ‘permissive annotation’ parameters (EC-weight=1, e-value-filter=0.1, GO-weight=5, HSP/hit coverage cut-off = 0%). In the third step of the annotation procedure, the pool of GO terms selected during the annotation step was merged with GO terms associated with the Interpro domain (InterproScan predictions based on the longest ORF). Finally, the Annex augmentation step was run to modulate the annotation by adding GO terms derived from implicit relationships between GO terms [46].

### Statistical analyses on libraries

We have used the randomization procedure (with 500 random datasets) and the R statistic, described in [47], to detect unigenes whose transcript abundance (number of ESTs) in symbiont-free and symbiont-full bacteriome libraries was statistically different (at a FDR of 5.5%). In order to perform a functional enrichment analysis of the unigenes extracted from the SSH, we used the Fatigo web tool [48] against the SO library.

### Transcriptomic study

#### Sample preparation

Transcriptomic analysis was performed on larval bacteriomes, whole symbiotic and aposymbiotic larvae, non-treated, mock-infected (injected with PBS), and injected with 10^5 ^*E. coli* (TOP10, Invitrogen, Cergy-pontoise, France). The *E. coli* bacterium was used here because it has been shown to efficiently induce the weevil immune system [6], and this bacterium does not necessitate an L2 safety lab structure for manipulation. Larvae were then maintained at 27.5°C and 70% rh for 6 hours. For each modality, 5 samples of 5 pooled larvae were prepared and then frozen at -80°C. Bacteriomes were dissected from non-treated larvae that have been maintained at 27.5°C and 70% rh for 6 hours. 5 samples of 25 pooled bacteriomes were dissected and then frozen at -80°C until RNA extraction.

#### Total RNA extraction and cDNA synthesis

Total RNA from whole larvae was extracted with the TRIzol Reagent (Invitrogen, Cergy-pontoise, France), following the manufacturer’s instructions. RNA was incubated with 1 U/g of RQ1 RNase-Free DNase (Promega, Charbonnières-les-Bains, France) for 30 min, at 37°C. Total RNA from bacteriomes was extracted with RNAqueous®-Micro (Ambion, Applied Biosystems, Austin, TX), which allows for a better RNA yield from small tissue samples. After purification, the RNA concentration was measured with a Nanodrop® spectrophotometer (Thermo Scientific, Wilmington, DE) and the RNA quality was checked on an agarose gel electrophoresis. Reverse-transcription into the first cDNA strand was carried out using the First strand Synthesis System for the RT-PCR kit (Invitrogen, Cergy-pontoise, France).

#### Real-time RT PCR transcript quantification

Quantitative measurements were performed on RNA samples originating from 5 independent replicates. Quantification was performed with a LightCycler®480 system using the LightCycler Fast Start DNA Master SYBR green I kit (Roche Diagnostics, Meylan, France). Data were normalized using the ratio of the target cDNA concentration to that of the glyceraldehyde 3-phosphate dehydrogenase (gapdh) gene and the ribosomal protein L29 (RPL29) gene. Primers were designed to amplify fragments with less than 250 bp and are listed in the additional file 1.

The PCR reactions were carried out in LightCycler 96-well plates, in a final volume of 10 μl, containing 2.5 μl of cDNA samples (diluted five-fold) and 7.5 μl of Light Cycler® 480 SYBR Green Master 1 mix, together with 0.5 μl of 10 mM of each primer, 1.5 μl H2O and 5 μl of Mastermix. Quantification was realized as described by [49]. Normalization and statistical pair-wise comparisons were determined using REST [50]. When comparing more than two modalities at the same time, the non-parametric Kruskal-Wallis test was used. RPL29 was shown to be the best housekeeping gene, with Bestkeeper tool [51], and this has been used in graphical representations.

## Results

### General characteristics of libraries: 8,941 weevil unigenes were generated

**Figure 2:**
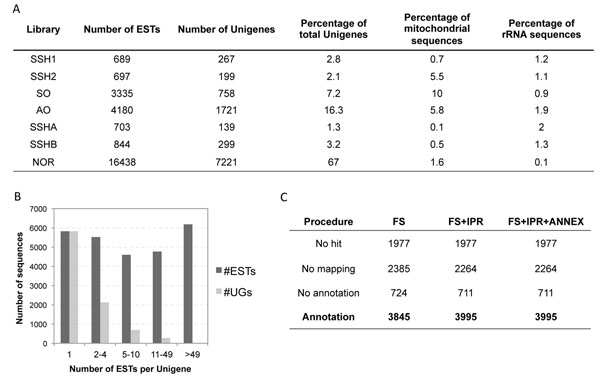
**General description of libraries.** (A) Table of ESTs and Unigene numbers presented for each library. The percentages of mitochondrial and rRNA sequences are also provided. (B) Distribution of unigenes (UGs) as a function of the number of ESTs involved in the UG sequences. UGs with only one EST are singletons, UGs with more than one EST are contigs. (C) Blast2go annotation results. Number of sequences presenting GO terms association is given for each step of the functional annotation. The different steps are described in the Methods section.

To explore bacteriome cellular specificities and weevil immune responses to bacteria, we have constructed 7 cDNA libraries from *S. oryzae* larvae. These libraries comprise the 4 SSH libraries, SSHA, SSHB, SSH1 and SSH2, the 2 non-normalized libraries from symbiont-full (SO) and symbiont–free (AO) bacteriomes and one normalized library (NOR) from whole aposymbiotic larvae challenged, and not, with *S. typhimurium* (Fig. 2A).

The sequencing of all the libraries has generated 26,886 readable ESTs with sequence mean lengths of 520 ± 177 bp. Contigation analysis has generated 8,941 unigenes. The average length of unigenes was 620 ± 260 bp, which suggests that most of the unigenes were obtained from low contigation of ESTs. Indeed, the analysis of unigene compositions in ESTs showed that about 88% of unigenes were obtained from between one (singleton) to four ESTs and less than 3.5% of unigenes were assembled from more than 10 ESTs (Fig. 2B). This finding highlights a low quantitative sequencing depth with the Sanger methodology and advocates next-generation sequencing (NGS) methods, such as Illumina, to fulfill *in silico* quantitative analysis of this work. The GC content of total sequences is about 35%, which is very close to the genomic GC content of *Tribolium castaneum* (34%), phylogenetically the closest Coleopteran species sequenced so far [52]. Sequences covered around 5.5 Mb against 14 Mb of predicted transcripts in *Drosophila*.

The distribution of unigenes in the different libraries is presented in Figure 2A. More than 60% of the unigenes were provided by the NOR library, showing the importance of normalization for unigene number enrichment. Blast analysis has shown that most of the first hits were from *Tribolium castaneum* sequences. This result was as expected and is linked with the relatively high phylogenetic proximity between *Tribolium* and *Sitophilus*.

Only about 25% of the unigenes had no Blast annotation that corresponded to the UTR part of the cDNA. Following the Blast2go annotation procedure for High Scoring Pair (HSP) coverage of 0%, 3845 unigenes presented at least one GO term (Fig. 2C). After Interproscan prediction and the Annex procedure, 3995 unigenes presented at least one GO term association.

### Analysis of libraries

One of the objects of this study was to unravel the genes involved in host-symbiont interactions within the bacteriome. For this purpose, an *in silico* subtraction was conducted between SO and AO libraries, which evaluates statistical differences in unigenes prevalence in the presence or absence of the symbiont in the bacteriome tissue. This analysis identified 11 differentially expressed genes (Table 2). The most differentially expressed gene showed the first blastx hit with a cellular Fatty-acid binding protein (FABP), and presented a calycin domain with the Interproscan tool. It is predicted that it would be upregulated in the presence of SPE. However, this first blastx hit presented a relative low e-value (*i.e.* 6e-05) and the predicted protein of the sequence showed a weak similarity with the fatty-acid protein (32% on 132 predicted amino acids). This finding highlights the need for additional work to clarify the annotation of this gene. As this gene was also reported as being the most highly expressed in the bacteriome of *S. zeamais *[30], it is referred to as the “Most Expressed Gene in the weevil Bacteriome” (MEGwB).

**Table 2:** List of unigenes presenting statistically different representations in AO and SO libraries.

*Accession numbers*	*^1^R*	*Unigene length* (*pb*)	*^2^Redundancy in AO*	*^2^Redundancy in SO*	*^3^1st blastx result on Tribolium Castaneum*	*^3^Accession*	*^3^ e-value*	*^3^Coverage*	*^3^Max identity*	*^4^InterProScan predicted domain*
FQ866673	16.45	664	24	103	allergen aca S13 (cellular FABP-like)	XP_969762	6e-05	50%	32%	IPR011038; IPR012674
FQ866935, FQ867818	15.91	1307	304	440	NA	NA	NA	NA	NA	no IPR
FQ877624	5.21	723	21	0	NA	NA	NA	NA	NA	No IPR
FQ884311	3.22	351	13	0	RPL37	XP_969650	3e-36	76%	94%	IPR001569; IPR011331; IPR011332; IPR018267
FQ868370	2.9	525	17	1	Chemosensory protein 10	NP_001039278	6e-33	75%	49%	IPR005055
FQ862292	2.9	974	17	1	Cathepsin L-like proteinase	NP_001163996	2e-68	88%	48%	no IPR
FQ869260	2.73	138	11	0	NA	NA	NA	NA	NA	No IPR
FQ865010	2.49	865	12	28	Gamma-subunit. methylmalonyl-CoA decarboxylase	XP_973308	2e-24	54%	58%	IPR010625
FQ884611, FQ867701	2.48	1463	10	0	Myoinositol oxygenase	XP_966469	3e-133	6%	74%	IPR007828
FQ864415	2.17	704	0	6	Transmembrane protein 41B	XP_975236	1.8e-02	25%	42%	No IPR
FQ863216	2.17	812	0	6	NA	NA	NA	NA	NA	no IPR

^1^The R statistic test, with 500 random datasets, was performed to evaluate genes whose representation in AO and SO libraries was statistically different. Sequences showing an R statistic > 2 were significant.

^2^Unigene redundancy is given for each library (AO and SO).

^3^For each unigene, we gave blastx matches with *Tribolium castaneum*, the closest genome-sequenced insect, phylogenetically, to *Sitophilus*. Accession numbers of *Tribolium* related sequences, e-value of blastx hits, sequences coverage and max identity between *Sitophilus* and *Tribolium* sequences are also given.

^4^Interproscan predicted domains are given to complete the characterization of sequences.

The subtraction has also identified two other sequences, which are highly expressed in the symbiont-full bacteriome, when compared to the symbiont-free bacteriome. The first was related to methylmalonyl-CoA decarboxylase (58% similarity based on predicted protein) and the second was a transmembrane protein close to the *Tribolium* transmembrane 41B protein. On the other hand, 4 sequences related to the cathepsin 1-like protein, the chemosensory protein, the ribosomal protein L37 and the myoinositol oxygenase, all showed significantly higher expression in the symbiont-free bacteriome. Finally, it is noteworthy that 4 sequences, including 2 more expressed in the symbiont-full bacteriome and 2 more expressed in the symbiont-free bacteriome, have neither Blast annotation nor an Interproscan definition domain. Such sequences cannot be used in this state and require further characterization.

In addition to *in silico* subtraction, SSHA and SSHB libraries were also constructed with the aim of identifying genes involved in host-symbiont interactions. As described in the Methods section, we carried out a functional enrichment analysis of SSHA and SSHB in order to highlight major GO terms associated with these library sequences (see Additional file 2). Concerning the SSHA library, three GO terms from biological processes (i.e. “transposition”, “cell division”, “DNA recombination”) and one GO term from molecular functions (i.e. “transposase activity”) were significantly over-represented. Concerning SSHB, five GO terms from biological processes (i.e. “digestion”, “nitrogen compound metabolic process”, “carbohydrate metabolic process”, “polysaccharide metabolic process”, and “energy derivation by oxidation of organic compounds”) and nine GO terms from molecular functions (i.e. “hydrolase activity”, “ion binding”, “tetrapyrole binding”, “hydrolase activity, acting on glycosyl bonds”, “monooxygenase activity”, “peptidase activity”, “heme binding”, “cation binding” and “hydrolase activity, hydrolyzing O-glycosyl compounds”) were significantly over-expressed.

The SSHA yielded 55 unigenes with the eukaryotic blast result. A detailed listing of these unigenes is presented in Additional file 3. The remaining unigenes were related to prokaryotic assignation, which means that the subtraction has been contaminated with symbiont DNA. Surprisingly, none of the 55 unigenes were related to the immune response and only one, an aspartic proteinase, presented a high similarity (96%) with a sequence found in *S. zeamais *[6]. Most of the SSHA unigenes are referred to as metabolic or cellular regulation genes, suggesting high cellular activity in the symbiont-full bacteriome [30]. The functional enrichment analysis has allocated, to the SSHA, the level 3 GO terms “transposition” (GO:0032196) and “transposase activity” (GO:0004803). This is probably due to the massive presence of insertion sequences (IS) recently documented in the SPE genome [17].

The 844 EST sequences from SSHB have provided 299 unigenes potentially expressed specifically in the symbiont-free bacteriome. Blastx annotations have identified around 60% of these sequences as digestive enzymes. Functional analysis of SSHB has allocated the level 3 GO terms, such as “digestion” (GO:0007586), “nitrogen compound metabolic process” (GO:0006807) or “hydrolase activity” (GO:0016787). As these functions are dominant in the gut tissue, and as symbiont-free bacteriomes are very thin, flat and intimately attached to the intestine, contamination from the gut is highly probable while dissecting out the bacteriomes.

### Transcriptomic study

The purpose of the transcriptomic study was to analyze molecular and cellular specificities of the bacteriome and to test the influence of symbiosis on the host immune response to bacterial pathogens. Analyzed genes were retrieved from different libraries based on *in silico* subtraction, experimental subtractions (SO, AO, SSHA), and on the examination of genes involved in cellular pathways of potential interest to intracellular symbiosis, such as apoptosis, cell trafficking and immunity (NOR, SSH1).

In total, we have selected 29 genes (Additional file 4). Except for MEGwB, all sequences presented more than 60% similarity with their first hit on the blastx and/or major Interproscan domains of the unigene predicted protein.

#### Differential gene expression in the bacteriome tissue

**Figure 3:**
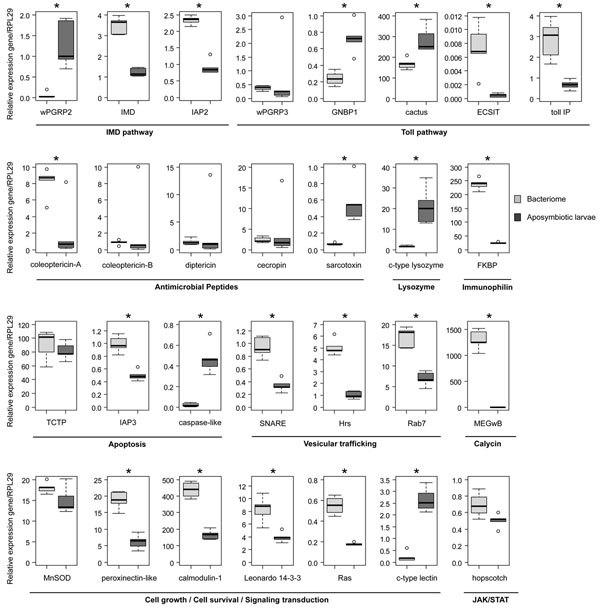
**Analysis of gene expression profiles in the bacteriome.** Transcripts of genes were quantified by qRT-PCR. Bacteriomes dissected from fourth-instar larvae were compared to whole aposymbiotic fourth-instar larvae. Expression of genes was normalized with the expression of the ribosomal protein L29. Each box represents the median (bolt line) and the quartiles (25% / 75%) of five independent measurements. Statistical analysis was performed with the REST pair-wise fixed reallocation randomization test. Asterisks indicate a significant difference between the bacteriome and the control (*p-value* < 0.05).

We have compared the steady state levels of 29 genes in the bacteriome and in the whole aposymbiotic larvae (Fig. 3). We preferred to use whole aposymbiotic larvae, rather than symbiont-free bacteriome tissue, as the control because SSHB is prone to a lot of potential contamination from the gut. The total transcriptome of larvae represented an average level of gene transcripts and this was then used as the control.

**Figure 4:**
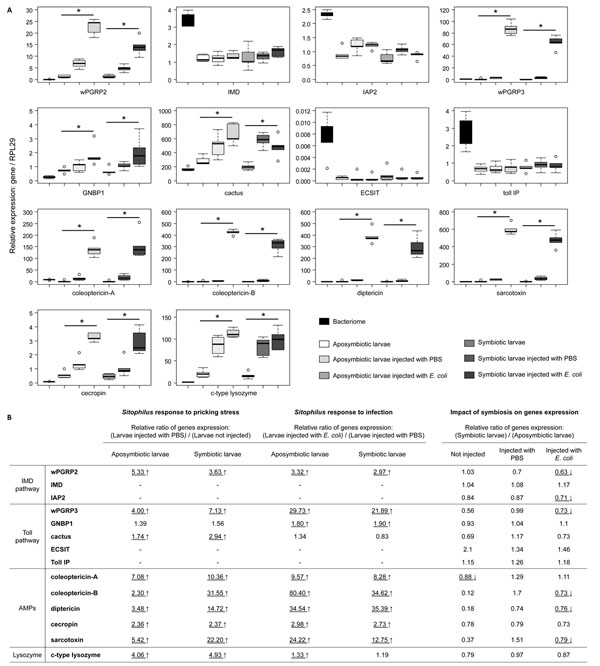
**Quantitative immune gene expression in symbiotic and aposymbiotic larvae of *Sitophilus oryzae*.** (A) Transcript levels of immune genes quantified by qRT-PCR in whole aposymbiotic and symbiotic larvae. For both symbiotic and aposymbiotic larvae, non-injected larvae, larvae injected with PBS, and larvae injected with *E. coli* were analyzed. Results from gene expression in the bacteriome are reported here as an indicator. Represented expression of genes was normalized with the expression of the ribosomal protein L29. Each box represents the median (bolt line) and quartiles (25% / 75%) of five independent measurements. For each symbiotic and aposymbiotic status, the non-parametric Kruskal-Wallis test was applied in order to determine global difference between the three modalities tested (*p-value* < 0.05), represented by an asterisk. (B) Differential expression ratios obtained from q-RT-PCR experiments. For genes presenting significant differences in expression after the global test (see A), the pricking stress effect was tested by comparing larvae injected, or not, with PBS. The infection effect was tested by comparing larvae injected with PBS and larvae injected with *E. coli*. The REST pair-wise fixed reallocation randomization test was applied. For each modality tested (not injected, injected with PBS and injected with *E. coli*), a comparison between symbiotic larvae and aposymbiotic larvae was applied in order to evaluate the impact of symbiosis on the expression of immune genes. The REST pair-wise fixed reallocation randomization test was performed between the expression of genes from symbiotic and aposymbiotic larvae. Underlined scores indicate significant differences between the two modalities tested (*p-value* < 0.05). An up-arrow indicates upregulated genes whereas a down-arrow indicates downregulated genes.

As described previously in *S. zeamais *[6], only Toll Interacting Protein (TollIP), as a potential negative regulator of the vertebrate Toll pathway [53] and coleoptericin-A, as AMP, are upregulated in the bacteriome of *S. oryzae*. The sarcotoxin and genes described as having lytic activity, such as *wpgrp2* (weevil PeptidoGlycan Recognition Protein2), *gnbp1* (Gram Negative Binding Protein1) and c-type lysozyme, are significantly down-regulated in the bacteriome when compared to aposymbiotic larvae challenged, or not, with *E. coli* (Fig. 3 and 4).

To gain a better understanding of immune regulation in the bacteriome, we have analyzed additional genes identified in this work, which are branched at different levels of the signaling pathways, including *imd* and *iap2* (IMD pathway), and *cactus* and *ecsit* (Toll pathway) [23,54-56]. Intriguingly, the *imd* and *iap2* genes, which activate AMP synthesis via the IMD pathway in *Drosophila*, are highly expressed in the *Sitophilus* bacteriome. Moreover, the *ecsit* gene, which participates in Toll-signaling pathway activation in vertebrates [56,57], is also highly expressed in the bacteriome whereas the Toll inhibitor *cactus* is downregulated (Fig. 3). Taken together, these data suggest that both IMD and Toll pathways are potentially initiated in the bacteriome, which appears to be in contrast with the low amounts of effector gene transcripts (e.g. AMP) in this tissue.

To extend this investigation to other cellular processes that are of interest to bacteriocyte homeostasis and survival, we have analyzed three genes potentially involved in apoptosis activation and regulation, namely the Inhibitor of APoptosis2 (*iap2*), the Inhibitor of APoptosis3 (*iap3*), and the caspase-like gene. Whilst apoptosis inhibitor genes (i.e. *iap2* and *iap3*) are highly expressed, the caspase-like encoding gene is weakly expressed in the bacteriome (Fig. 3 and 4). In line with this finding, the RAt Sarcoma (*Ras*), *calmodulin-1* and *leonardo 14-3-3*, which are all involved in cell growth and survival [58-60], are also upregulated in the bacteriome. Taken together, these data suggest that bacteriocyte cell pathways are regulated to prevent cell death and to promote cell survival.

Vesicular trafficking is also an important process in the bacteriocyte functions, both for metabolic exchange between the host and the endosymbiont [30] and for intracellular bacterial control by cellular autophagy [61]. Among the selected genes, we have tested three genes involved in vesicular formation and trafficking, these being the Ras related GTP-binding gene (*Rab7*, late endosomes labelling), the hepatocyte growth factor-regulated tyrosine kinase substrate (*Hrs*, involved in endosomal maturation) and a gene encoding for a Soluble NSF Attachment protein REceptor (SNARE, vesicle fusion) [62-64]. We have demonstrated that all these genes are highly expressed in the bacteriome, when compared to the aposymbiotic larvae (Fig. 3).

Finally, the most highly represented gene transcript in the bacteriome is MEGwB (more than 1500 fold, compared to aposymbiotic larvae). While this high expression suggests an important role for this gene in relation to symbiosis, bioinformatic analysis did not, unfortunately, determine precisely the function of this gene. Nevertheless, the MEGwB sequence includes a calycin domain that characterizes lipocalins and FABP genes. Lipocalins have been shown to be modulators of the immune response in vertebrates [65,66], and an FABP protein has been seen to be active in cell proliferation caused by tumors [67].

#### Influence of symbiosis on host immune gene expression

In order to test whether the insect immune response to bacterial pathogens is influenced by symbiosis, we have compared immune gene expression between symbiotic and aposymbiotic larvae. We have analyzed both larval responses to pricking stress (PBS injection) and to the challenge of the Gram-negative bacterium, *E. coli* (Fig. 4).

Both symbiotic and aposymbiotic larvae were shown to respond slightly, but significantly, to an injection of PBS in the hemolymph. Induced genes included *wpgrp2*, *wpgrp3*, *gnbp1*, *cactus*, c-type lysozyme and all AMPs. When larvae were challenged with *E. coli*, all of these genes (except *cactus* and c-type lysozyme) were highly induced, when compared with the mock-infected larvae.

Concerning the impact of symbiosis on immune response efficiency, the stress generated by PBS injection did not induce any significant difference between symbiotic and aposymbiotic larvae at the transcriptional level for all the genes studied. However, following infection with *E. coli*, aposymbiotic larvae displayed a higher expression of immune gene, when compared with symbiotic larvae (Fig. 4). Among the genes studied, *wpgrp2*, *wpgrp3*, the coleoptericin-B, the sarcotoxin and the diptericin were all significantly less induced in symbiotic insects than in aposymbiotic ones.

## Discussion and conclusion

The last decade has seen a growing number of projects investigating the molecular and cellular interactions between invertebrate hosts and their symbionts [5-7,30,68-73]. These have focused on the immune (and bacterial) adaptive changes that favor the establishment of symbiosis [18,70], the maintenance and control of symbiosis [6,72,74,76], and the impacts of symbiosis on host immunocompetence and fitness benefit [9,77-82]. While recent data have provided original and exciting insights in these fields, much more effort needs to be deployed on the molecular and genetic aspects of additional invertebrate systems to unravel the conserved and diverged mechanisms in host-symbiont interactions. With this aim, we have first enlarged the gene repertoire of the cereal weevil *S. oryzae* and, secondly, we have used qRT-PCR to examine the expression of a set of genes in different conditions, taking into consideration the bacteriocyte molecular basis and symbiont impacts on the host immune response.

Bioinformatic analyses of 26,886 EST sequences, from different libraries, have generated 8,941 unigenes. This gene repertoire, along with the recent effective application of RNA interference (RNAi) technology in *Sitophilus *[49], will enable us to carry out more functional studies and to decipher cellular mechanisms that underlie long-term symbiont persistence, and bacteriocyte homeostasis and maintenance. Nevertheless, while the Sanger sequencing methodology has significantly enhanced unigene number in *S. oryzae*, additional NGS needs to be realized in order to accurately analyze the transcriptome quantitatively, and to decipher the functions of interest to symbiosis at gene level.

As regards symbiont persistence, we have previously reported that one insect strategy to maintain long-term relationships with endosymbionts consists of compartmentalization of the bacteria into the bacteriocyte cells, which exhibit a local and structured immune response to tolerate the endosymbiont [6]. Indeed, while the experimental injection of the endosymbiont into the weevil hemolymph resulted in a drastic induction of genes encoding immune effectors, only a few immune genes were upregulated in the bacteriome, including the *wpgrp1* and the *Tollip* that are homologs to genes described as immune modulators [6,53,83]. The former is a homolog of the dipteran *pgrp-lb* gene, the expression of which downregulates the IMD pathway [76,84], and the latter was suspected of being a negative regulator of the vertebrate Toll pathway [53]. To gain a better insight into how IMD- and Toll-like pathways are regulated in the bacteriome tissue, we have examined the expression of additional genes identified in this work, which are branched at different levels of the signaling pathways. As a result, genes involved in the activation of IMD- and Toll-like pathways (i.e. *imd*, *iap2*, and *ecsit*) were highly expressed in the bacteriome, whereas the inhibitor *cactus* gene exhibited the opposite profile, which suggests that the IMD- and Toll-like pathways may potentially be activated in the *Sitophilus* bacteriome. This finding is initially intriguing since the end products of these pathways (i.e. the AMPs) are either absent or only weakly expressed in the bacteriome. However, taking into consideration that the Toll gene was first described as an essential component in establishing the dorsoventral axis in *Drosophila* embryo [85], and that IMD is connected with other cellular pathways, such as apoptosis [86], it is possible that IMD- and Toll-like pathways may be involved in developmental processes and in the homeostasis of symbiotic tissues. Such an assumption is supported by a similar immune pattern (i.e. high expression of Toll and low expression of AMPs) reported for the mutualistic association between *Wolbachia* and the parasitoid wasp, *Asobara tabida *[36]. However, the reason for the high expression of coleoptericin-A in the bacteriocyte is still unexplained. Whether IMD- and/or Toll-like pathways are branched on the coleoptericin-A synthesis pathway remains to be clarified from further investigations. Finally, while IMD- and Toll- like pathways seem to be activated in the bacteriocyte, it is possible that the inhibition of signal transduction by gene regulators is involved. For instance, *wpgrp1* and *tollip* genes are good regulator candidates and they could play a crucial role in this inhibition [76,84]. Recently, Ryu et *al*. [75] have reported that the *Drosophila* homeobox gene *caudal* also regulates the commensal-gut bacteria by repressing the nuclear factor Kappa B-dependent AMP genes. Ongoing RNAi experiments will provide more information about the function and the regulation of these pathways in the *Sitophilus* system.

The high accumulation of transcripts from *Rab7*, *Hrs* and *SNARE* genes could be viewed as being due to intense endosomal trafficking within the bacteriocyte. These genes are certainly very involved in vesicle synthesis and fusion [62-64]. Moreover, intense vesicle trafficking has already been observed by electronic microscopy within *Sitophilus* bacteriocytes [30]. Vesicle trafficking may aid in metabolic component exchanges between the host and the symbiont, or it may help in endosome fusion, with late endosomes and lysozomes, to favor autophagy. For the latter, we can speculate about the possibility that autophagy could serve as an additional host mechanism to regulate symbiont density. In support of this hypothesis, *in silico* cDNA comparison between symbiont-full and symbiont-free ovaries has shown that vesicle trafficking is also highly represented in the presence of *Wolbachia* in the isopod *Armadillidium vulgare *[35]. Moreover, receptors of innate immunity have been identified on vertebrate endosome membranes [57,87] and autophagy has been described as a possible means of eliminating intracellular pathogens [61].

To permanently sequester the endosymbiont within the bacteriome, and to avoid bacterial invasion into insect tissues, bacteriocyte cells need to maintain homeostasis and to survive during insect developmental stages. While apoptosis has been observed as a response to infection by a wide range of animal and plant pathogens [88,89], very limited data are available on invertebrate symbiotic systems [70]. To tackle this question in the *Sitophilus* system, we have analyzed genes potentially involved in apoptosis inhibition (*iap2* and *iap3*) and apoptosis execution (caspase-like). We have shown that the high expression of apoptosis inhibitor genes paralleled the low amount of caspase-like gene transcripts in the bacteriome. In addition to the upregulation of genes involved in cell growth, such as *Ras* and *leonardo* 14-3-3, these preliminary data suggest that weevil bacteriocytes manage to survive an endosymbiont infection by inhibiting the apoptosis pathway. Inhibition of apoptosis can also be mediated by the expression of the FK506BP gene (or FKBP). In vertebrates, the FKBP38 gene inhibits apoptosis by interacting with Bcl-2 [90]. Moreover, we cannot exclude the possibility that apoptosis inhibition is manipulated by the symbiont for its own survival. Such a mechanism has been described in *Asobara tabida*, where *Wolbachia* elimination with antibiotic treatment led to the activation of apoptosis in female ovaries [5].

A striking result of this current study was that symbiotic larvae presented a lower immune response to bacterial challenge, when compared to aposymbiotic larvae.

Invertebrate immune reactions toward pathogens, and the possible evolutionary impact of endosymbiosis on shaping these reactions, have been the major focus of research in the past few years [69,73,77,79-81]. The recent genome sequencing of the pea aphid, which shares a long-term symbiotic relationship with the endosymbiont *Buchnera*, has surprisingly revealed that aphids lack crucial components of the IMD pathway [73]. Furthermore, no apparent AMP was determined by gene annotation [73,91]. In the same context, Braquart-Varnier et *al*. [77] have shown that the cellular immune response could be affected by endosymbionts. Isopods harboring *Wolbachia* (wVulC) exhibited lower haemocyte density and more intense septicaemia in the haemolymph. In the ant, *Camponotus fellah*, insect treatment with the Rifampin antibiotic resulted in a drastic decrease in the number of symbiotic bacteria, and this decrease was associated with a higher encapsulation rate when compared with the non-treated insect control [92]. Diminished encapsulation ability in parasitoid *Leptopilina* eggs has also been reported, in the presence of *Wolbachia*, in *D. simulans *[93]. Taken together, these findings lead to the hypotheses that either invertebrate symbiosis may have selected for a simplification of the host immune system or endosymbionts manage to modulate the host immune expression, presumably for their own survival. A third hypothesis is that invertebrates might allocate different resources to immune pathways. In this case, the relatively low systemic response in weevil symbiotic larvae could be due to the allocation of insect resources to local expression of the bacteriome, to the detriment of the humoral systemic expression.

However, although these hypotheses appear to be compatible with our preliminary results on *Sitophilus*, additional work needs to be done to determine whether decreases in AMP gene expression in symbiotic insects are due to endosymbiont manipulation or whether heat-treatment while obtaining apsoymbiotic insects has resulted in a genetic selection of host immunocompetence. Moreover, it is notable that the endosymbiosis interaction with the invertebrate immune system is an emerging field that provides quite contrasting data. Contrary to previous findings, several studies investigating *Wolbachia* as a potential control agent in vector insect species have reported that *Wolbachia* can activate the host immune system, and protect the insect against a wide variety of pathogens [79-82]. However, as only a few *Wolbachia* strains have been tested so far (i.e. *w*MelPop and *w*AlbB), and since many experiments were conducted on a heterologous host system, further investigations are needed to reveal whether insect immune activation is limited to some host-*Wolbachia* systems, or whether multiple strategies are being used by endosymbionts to ensure their own survival and to help their host to survive any pathogens.

In conclusion, this work provides a large repertoire of *S. oryzae* EST coding sequences that will help in future molecular and functional investigations, both into symbiosis and other topics related to insect physiology and development. Transcriptomic analyses have elucidated the bacteriome local immune response and indicated new cellular regulations of potential interest in intracellular symbiosis. Moreover, data provided on host immunocompetence variations in relation to symbiosis broaden and reinforce the idea that invertebrate symbiotic associations may have shaped some host immune functions. This work should stimulate further genetic and functional studies to determine how immunity is modified to accommodate the symbiont partner and how endosymbionts manipulate the immune response for their own survival and to enable the host to resist pathogens.

## Authors' contributions

AV designed and performed experiments, analyzed data (statistics and bioinformatics), wrote the paper and participated in bioinformatic analysis; DC set up the bioinformatic tools and analyzed all the libraries and EST sequences; CVM participated in the construction of the libraries and the molecular study, performed the insect challenge experiment with *Salmonella* and performed RNA extraction; AVa carried out dissections and qRT-PCR; FG and PW realized EST sequences; AH conceived the study, coordinated the work and helped to draft and write the manuscript.

All authors have read and approved the final manuscript.

## Competing interests

The authors declare that they have no competing interests.

## Supplementary Material

Additional file 1 Primers used for transcriptomic study Click here for file

Additional file 2 Results of functional enrichment on SSHA and SSHB Click here for file

Additional file 3 Eukaryotic sequences generated in SSHA Click here for file

Additional file 4 Characteristics of sequences used in transcriptomic study Click here for file
