# Supplementary material for: Host gene response to endosymbiont and pathogen in the cereal weevil Sitophilus oryzae
Source: BMC Microbiol. 2012 Jan 18;12(Suppl 1):S14. doi: 10.1186/1471-2180-12-S1-S14 (PMC3287511; doi:10.1186/1471-2180-12-S1-S14)
Supplement: Additional file 1 — Primers used for transcriptomic study [file 1471-2180-12-S1-S14-S1.pdf]

Additional file 1 – Primers used for transcriptomic study

qRT-PCR Primers	Sequence (5′-3′)	T°C (4GC+2AT)
For-MEGwB	GCCAAAACCAAAGCCACTTA	58
Rev-MEGwB	TGTCGGGACTCCTTTCTTTG	60
For-Hrs	AGTCAGAGGCTGAAGCCAAG	62
Rev-Hrs	GATGGAGCTGAAGGGCTAGT	62
For-coleoptericin-A	GAATAGATACAACGGGGGTCA	62
Rev-coleoptericin-A	CTACCATCTGACACTTCCTC	60
For-coleoptericin-B	CAGTTACATTTCGCCCCCTA	60
Rev-coleoptericin-B	GGCTGCTGGGTCCTCTAATA	62
For-SNARE	CCCTGACTGCGGATATCACT	62
Rev-SNARE	TAATCGCTTCAATTCAGCCC	58
For-Rab7	TCGAGGAGCAGACTGTTGTG	62
Rev-Rab7	GCCCTTTTTGCACTGATAGC	60
For-Peroxinectin	GAGCTTTTTGCGGTTTGAAG	58
Rev-Peroxinectin	GTCCTTGCCATTTGTTCGTT	58
For-TCTP	AGTCAGCTCTTCACGGAGGT	62
Rev-TCTP	TGGCCCCATCAAGTAAAATC	58
For-Leonardo 14-3-3	GCTTAGGGAGATATGCGACG	62
Rev-Leonardo 14-3-3	ACTGCATTTTTGGACTTGGC	58
For-Hopscotch	ACAGTTTACCGGTACCAGCG	62
Rev-Hopscotch	TACTTGTTGCTCCTGCCCTT	60
For-c-type lectin	ATAGCCGTAGTTGCCACACC	62
Rev-c-type lectin	ACAAAGATTCCTGCCATCCA	58
For-Calmodulin1	GGTAACGGAACGATCGACTT	62
Rev-Calmodulin1	CCTGATCATCTCGTCCACCT	60
For-FK506BP	ATTAATCCCGGAGACGGTTC	60
Rev-FK506BP	CGACACTCATCTGGGCTAC	60
For-C-type lysozyme	TCCTTGGACCAGTTAGGCAC	62
Rev-C-type lysozyme	TGCGCACTTTATGTCTCCA	56
For-cactus	GGCGACGAGAATATCCTGAG	62
Rev-cactus	CATCTCTTCCTCGTCTTCGC	62
For-GNBP1	CTGGATTTAACTCGCCAAGA	58
Rev-GNBP1	ACTGTCTTTCCAAGAGGGCA	60

qRT-PCR Primers	Sequence (5′-3′)	T°C (4GC+2AT)
For-IAP3	TCATGACAAAGGGACAATGC	58
Rev-IAP3	TGAAAACCTTCGATTCCTGG	58
For-Imd	ACAGGAGCAGTTTTCGTTCG	60
Rev-Imd	GTTCAACCTTTGGGGTTCC	58
For-Ras	AATTGGTTGTTGTGGGTGCT	58
Rev-Ras	TGATCACGCATTGCACTGT	56
For-caspase	TCCAGGAACGACATCATTTG	60
Rev-Caspase	TCGGCACTGTAATCCGTACA	60
For-ECSIT	TTTTCCACACGTCTGCGTTA	58
Rev-ECSIT	CTGAACTCCGAACTCTTCCG	62
For-MnSOD	ATGATTATGCCGCTTTGGAG	58
Rev-MnSOD	TTAATGTGTCCCCCACCATT	58
For-IAP2	GGACAATGCAGAGCAGACAA	60
Rev-IAP2	GCGTCTGGTTGAGAATAGC	58
For-gapdh	AACTTTGCCGACAGCCTTGG	62
Rev-gapdh	GCGCCCATGTATGTAGTTGG	62
For-RPL29	TGGCCAAGTCCAAGAATCACA	62
Rev-RPL29	TTCTTGGCGCTAGCTTGTCTT	62
For-diptericin	CTCCGATTTCAAGCCGACAG	62
Rev-diptericin	GGACCGTTTGACCATACTGG	62
For-cecropin	GTCCTGTTAGTTGTGGCAGTA	62
Rev-cecropin	TTATCTTCCAGGCACCAATCC	62
For-sarcotoxin	AGTCACAAAGGAACAATTTTGG	60
Rev-sarcotoxin	AGAAGCCGTCACGTCCGTTC	64
For-wPGRP2	TGCAAGGCCTGCCCTTAGT	60
Rev-wPGRP2	GTGCCATCTCCTCCAATCAT	60
For-wPGRP3	TTATGAAGGAGCTGGATGGC	60
Rev-wPGRP3	TAACTTGCCGACCTCCAAAC	60
For-Tollip	AAGAGCGACGCAATAGGGT	58
Rev-Tollip	CATTAGGGTCCGTAGGTGT	58
