# Supplementary material for: Host gene response to endosymbiont and pathogen in the cereal weevil Sitophilus oryzae
Source: BMC Microbiol. 2012 Jan 18;12(Suppl 1):S14. doi: 10.1186/1471-2180-12-S1-S14 (PMC3287511; doi:10.1186/1471-2180-12-S1-S14)
Supplement: Additional file 2 — Results of functional enrichment on SSHA and SSHB [file 1471-2180-12-S1-S14-S2.pdf]

Additional file 2 – Results of functional enrichment on SSHA and SSHB

SSHA *vs*. SO

**Biological process**			
Level	GO terms	p-value	Adjusted p-value
3	transposition (GO:0032196)	1.60E-04	0.0114
	cell division (GO:0051301)	1.42E-03	0.0504
6	DNA recombination (GO:0006310)	1.60E-04	0.0696
**Molecular function**			
3	transposase activity (GO:0004803)	1.50E-04	0.0124

SSHB *vs*. SO

**Biological process**			
Level	GO terms	p-value	Adjusted p-value
3	digestion (GO:0007586)	5.40E-04	0.0381
	nitrogen compound metabolic process (GO:0006807)	0.018	0.0383
4	carbohydrate metabolic process (GO:0005975)	2.60E-06	4.40E-04
5	polysaccharide metabolic process (GO:0005976)	1.40E-04	3.41E-03
	energy derivation by oxidation of organic compounds (GO:0015980)	2.50E-04	0.0405
**Molecular function**			
3	hydrolase activity (GO:0016787)	1.90E-10	1.60E-08
	ion binding (GO:0043167)	2.20E-04	7.05E-03
	tetrapyrole binding (GO:0046906)	2.50E-04	7.05E-03
4	hydrolase activity. acting on glycosyl bonds (GO:0016798)	2.80E-05	4.99E-03
	monooxygenase activity (GO:0004497)	7.30E-05	6.55E-03
	peptidase activity (GO:0008233)	1.70E-04	0.14
	heme binding (GO:0020037)	0.011	0.0453
	cation binding (GO:0043169)	2.60E-03	0.093
5	hydrolase activity. hydrolyzing O-glycosyl compounds (GO:0004553)	6.40E-05	0.0188
