# Supplementary material for: Host gene response to endosymbiont and pathogen in the cereal weevil Sitophilus oryzae
Source: BMC Microbiol. 2012 Jan 18;12(Suppl 1):S14. doi: 10.1186/1471-2180-12-S1-S14 (PMC3287511; doi:10.1186/1471-2180-12-S1-S14)
Supplement: Additional file 3 — Eukaryotic sequences generated in SSHA [file 1471-2180-12-S1-S14-S3.pdf]

Additional file 3 – Eukaryotic sequences generated in SSHA

Accesion numbers	#ESTs	Length (pb)	description	Species	Accession	Coverage	e-value	Max identity
FQ859854, FQ859644	2	288	similar to scavenger receptor class B (AGAP005716-PA)	*Tribolium castaneum*	XP_970008	46%	0.028	37%
FQ859499, FQ859446	2	194	NA	NA	NA	NA	NA	NA
FQ859780, FQ859427	2	150	NA	NA	NA	NA	NA	NA
FQ859273, FQ859248	2	164	similar to 6-phosphogluconate dehydrogenase	*Tribolium castaneum*	XP_972051	96%	3.00E-18	81%
FQ859351, FQ859330	2	437	calmodulin-1	*Trichinella spiralis*	EFV60806	61%	1.00E-44	100%
FQ859529, FQ859251	2	410	calmodulin-1	*Trichinella spiralis*	EFV60806	91%	1.00E-63	100%
FQ859762, FQ859397	2	384	similar to mitochondrial intermembrane space import and assembly protein 40	*Tribolium castaneum*	XP_967514	89%	9.00E-43	73%
FQ859589, FQ859345	2	347	tubulin alpha-1 chain	*Trichinella spiralis*	EFV54402	99%	3.00E-58	99%
FQ859824, FQ859776, FQ859840	3	676	similar to AGAP003323-PA Kinesin 2C	*Tribolium castaneum*	XP_971861	10%	0.022	75%
FQ859795, FQ859681, FQ859724, FQ859382, FQ859461, FQ859671, FQ859368	7	397	similar to Transmembrane protein 41B	*Tribolium castaneum*	XP_975236	32%	0.53	43%
FQ859737, FQ859455, FQ859532, FQ859493, FQ859259, FQ859488, FQ859594, FQ859869	8	810	similar to anillin, actin binding protein	*Monodelphis domestica*	XP_001374467	99%	4.00E-23	28%
FQ859189	1	155	nanchung	*Tribolium castaneum*	EFA04638	98%	4.00E-19	90%
FQ859195	1	467	similar to AGAP006254-PA	*Tribolium castaneum*	XP_967579	99%	2.00E-70	85%
FQ859215	1	689	similar to pyrazinamidase/nicotinamidase	*Tribolium castaneum*	XP_973348	99%	6.00E-94	66%
FQ859227	1	312	similar to AGAP012394-PA	*Tribolium castaneum*	XP_967272	99%	6.00E-40	70%
FQ859228	1	178	GI22356	*Drosophila mojavensis*	XP_002000741	97%	1.00E-13	63%
FQ859256	1	591	similar to CG3279-PA	*Tribolium castaneum*	XP_970611	90%	1.00E-75	82%
FQ859261	1	157	similar to proteasome beta subunit	*Tribolium castaneum*	XP_968855	97%	2.00E-19	88%
FQ859289	1	712	Retrovirus-related Pol polyprotein from transposon 412	*Drosophila melanogaster*	P10394	52%	1.00E-11	33%
FQ859294	1	287	NA	NA	NA	NA	NA	NA
FQ859296	1	278	similar to CG1972 CG1972-PA	*Tribolium castaneum*	XP_969343	31%	4.00E-05	75%
FQ859298	1	148	hypothetical protein EAG_03557	*Camponotus floridanus*	EFN71375	97%	8.00E-18	81%
FQ859320	1	284	similar to MYC binding protein 2	*Tribolium castaneum*	XP_971508	99%	1.00E-26	64%
FQ859341	1	136	hypothetical protein TcasGA2_TC013494	*Tribolium castaneum*	EFA03494	59%	9.00E-04	77%
FQ859343	1	193	NA	NA	NA	NA	NA	NA
FQ859369	1	199	similar to sugar transporter	*Tribolium castaneum*	XP_967393	73%	4.00E-08	53%
FQ859372	1	296	similar to mitochondrial ATP synthase coupling factor 6	*Tribolium castaneum*	XP_969272	86%	3.00E-19	62%
FQ859387	1	556	similar to CG1440 CG1440-PC	*Tribolium castaneum*	XP_001814047	37%	5.00E-20	74%
FQ859400	1	102	similar to trehalase	*Tribolium castaneum*	XP_973976	94%	1.00E-05	65%
FQ859404	1	457	Centromere protein X	*Bos taurus*	NP_001107999	49%	9.00E-05	58%
FQ859418	1	231	aspartic proteinase	*Sitophilus zeamais*	BAH24176	98%	6.00E-34	96%
FQ859423	1	213	similar to epoxide hydrolase-related	*Tribolium castaneum*	XP_975099	98%	3.00E-15	49%
FQ859428	1	145	GTP-binding protein YPTC1	*Trichinella spiralis*	EFV59881	99%	4.00E-19	97%
FQ859436	1	439	GL27105	*Drosophila persimilis*	XP_002027924	38%	1.00E-09	54%
FQ859440	1	704	similar to CG8306 CG8306-PA	*Tribolium castaneum*	XP_973467	99%	6.00E-89	67%
FQ859457	1	318	hypothetical protein TcasGA2_TC011527	*Tribolium castaneum*	EFA11369	76%	7.00E-22	60%
FQ859474	1	603	similar to zipper CG15792-PD myosin heavy chain 2	*Tribolium castaneum*	XP_974183	99%	5.00E-111	97%
FQ859478	1	540	hypothetical protein TcasGA2_TC014935	*Tribolium castaneum*	EFA04876	99%	2.00E-78	81%
FQ859480	1	430	similar to restin (Reed-Steinberg cell-expressed intermediate filament-associated protein)	*Tribolium castaneum*	XP_967018	93%	3.00E-56	81%
FQ859503	1	118	similar to RH56418p	*Tribolium castaneum*	XP_967345	99%	9.00E-12	92%
FQ859526	1	485	similar to mandelate racemase	*Tribolium castaneum*	XP_972041	89%	1.00E-62	77%
FQ859554	1	675	hypothetical protein TcasGA2_TC006365	*Tribolium castaneum*	EFA08694	60%	7.00E-25	60%
FQ859576	1	547	elongation factor 1-alpha	*Sitophilus oryzae*	AAN08765	99%	9.00E-97	95%
FQ859587	1	135	similar to presqualene diphosphate phosphatase	*Tribolium castaneum*	XP_972110	97%	5.00E-13	68%
FQ859682	1	688	similar to AGAP006818-PA	*Tribolium castaneum*	XP_969259	90%	3.00E-91	79%
FQ859696	1	113	similar to dynein light intermediate chain	*Tribolium castaneum*	XP_968528	87%	2.00E-06	78%
FQ859704	1	375	similar to glutathione peroxidase	*Tribolium castaneum*	XP_969937	84%	2.00E-53	91%
FQ859733	1	372	similar to FK506-binding protein-like protein	*Tribolium castaneum*	XP_969563	82%	3.00E-49	88%
FQ859746	1	177	similar to CG16708 CG16708-PA	*Tribolium castaneum*	XP_969216	71%	2.00E-10	73%
FQ859772	1	237	similar to S-adenosylmethionine synthetase isoform 1	*Tribolium castaneum*	XP_966678	98%	1.00E-36	87%
FQ859777	1	268	similar to AGAP004510-PA	*Tribolium castaneum*	XP_001809545	98%	1.00E-38	80%
FQ859781	1	252	similar to AGAP002752-PA	*Tribolium castaneum*	XP_969161	98%	4.00E-23	59%
FQ859794	1	256	similar to AGAP010451-PA	*Tribolium castaneum*	XP_973021	96%	1.00E-29	79%
FQ859812	1	106	similar to plasma glutamate carboxypeptidase	*Acyrthosiphon pisum*	XP_001951862	99%	0.083	57%
FQ859814	1	355	similar to thyroid hormone receptor interactor 12 isoform 1	*Tribolium castaneum*	XP_966614	99%	3.00E-56	95%
