# Supplementary material for: Host gene response to endosymbiont and pathogen in the cereal weevil Sitophilus oryzae
Source: BMC Microbiol. 2012 Jan 18;12(Suppl 1):S14. doi: 10.1186/1471-2180-12-S1-S14 (PMC3287511; doi:10.1186/1471-2180-12-S1-S14)
Supplement: Additional file 4 — Characteristics of sequences used in transcriptomic study [file 1471-2180-12-S1-S14-S4.pdf]

Additional file 4 – Characteristics of sequences used in transcriptomic study

Accesion numbers	Reference name	Sequence length (pb, based on the longest ORF)	1st blastx hit	Related species	Accession number	e-value	Coverage	Max identity	Interproscan domains
FQ872200, FQ873253, FQ878329	wPGRP2	591	peptidoglycan recognition protein 2	*Sitophilus zeamais*	ABZ80671	5E-103	94%	97%	IPR002502, IPR006619, IPR015510
FQ872675	IMD	519	similar to immune deficiency CG5576-PA	*Tribolium castaneum*	XP_971829	3E-23	100%	39%	IPR000488, IPR011029
FQ882813	IAP2	585	similar to inhibitor of apoptosis 2 protein	*Tribolium castaneum*	XP_975027	9E-46	98%	55%	IPR001370
FQ861391	wPGRP3	582	similar to Tm-peptidoglycan recogntition protein-SA	*Tribolium castaneum*	XP_969883	2E-64	89%	58%	IPR002502, IPR006619, IPR015510, IPR017331
FQ868488	GNBP1	384	GNBP1	*Tenebrio molitor*	BAG14263	5E-24	97%	40%	IPR000757, IPR008985, IPR013320
FQ874505, FQ879166, FQ872774	cactus	432	cactus isoform 1	*Tribolium castaneum*	NP_001157183	1E-24	72%	51%	IPR002110, IPR020683
FQ871672	ECSIT	363	similar to Evolutionarily conserved signaling intermediate in Toll pathway, mitochondrial	*Tribolium castaneum*	XP_970998	2E-18	75%	66%	IPR010418
FQ872701	Toll IP	600	tollip-like protein	*Sitophilus zeamais*	ABZ80666	5E-98	100%	98%	IPR000008, IPR008973
FQ880455	coleoptericin-A	378	coleoptericin 1	*Sitophilus zeamais*	ABZ80661	1E-57	99%	91%	IPR009382
FQ861122	coleoptericin-B	348	coleoptericin 2	*Sitophilus zeamais*	ABZ80667	2E-54	98%	96%	IPR009382
FQ870576	diptericin	402	hypothetical antimicrobial peptide	*Sitophilus zeamais*	ABZ80662	1E-53	99%	93%	IPR005521
FQ870561	cecropin	210	hypothetical antimicrobial peptide	*Sitophilus zeamais*	ABZ80668	2E-28	98%	91%	noIPR
FQ882365	sarcotoxin	588	hypothetical antimicrobial peptide	*Sitophilus zeamais*	ABZ80663	3E-90	92%	97%	IPR005521
FQ878880	c-type lysozyme	441	c-type lysozyme	*Sitophilus zeamais*	ABZ80664	9E-78	99%	97%	IPR001916, IPR023346
FQ880527	TCTP	612	putative translationally controlled tumor protein	*Maconellicoccus Hirsutus*	ABM55515	2E-66	84%	79%	IPR011057, IPR011323, IPR018103, IPR018105
FQ876170	IAP3	366	inhibitor of apoptosis	*Spodoptera littoralis*	CAM96614	4E-14	72%	55%	IPR001370
FQ872562	caspase-like	579	caspase-like protein	*Tribolium castaneum*	NP_001164145	2E-24	95%	36%	IPR001309, IPR002398, IPR011600
FQ875197, FQ871532	MnSOD	645	similar to Mn superoxide dismutase	*Tribolium castaneum*	XP_972440	2E-88	99%	70%	IPR001189, IPR019831, IPR019832, IPR019833
FQ880385, FQ871991, FQ877260	peroxidase, peroxinectin-like	585	peroxidase	*Tribolium castaneum*	NP_001164313	1E-56	92%	55%	IPR002007
FQ859529	calmodulin-1	410	calmodulin-1	*Trichinella spiralis*	EFV60806	1E-63	91%	100%	IPR002048, IPR011992, IPR018247, IPR018248, IPR018249
FQ863830	Leonardo 14-3-3	534	similar to 14-3-3 CG17870-PA, isoform A isoform 2	*Apis melifera*	XP_623183	2E-80	97%	93%	IPR000308, IPR023409, IPR023410
FQ884691	Ras	285	Ras-like protein 1	*Harpegnathos saltator*	EFN88166	9E-39	97%	97%	IPR001806, IPR003577, IPR003578, IPR003579, IPR013753, IPR020849
FQ882388, FQ871450	c type lectin	696	similar to CG3244 CG3244-PA isoform 1	*Tribolium castaneum*	XP_967043	6E-120	92%	90%	IPR001304, IPR016186, IPR018378
FQ883872	hopscotch	408	hopscotch	*Tribolium castaneum*	EFA07411	6E-47	94%	71%	IPR000719, IPR001245, IPR008266, IPR011009, IPR020635
FQ875934, FQ871575	SNARE	651	similar to CG3279 CG3279-PA	*Tribolium castaneum*	XP_970611	5E-78	99%	74%	IPR007705
FQ874490, FQ882701	Hrs	834	similar to hepatocyte growth factor-regulated tyrosine kinase substrate	*Tribolium castaneum*	XP_967857	4E-75	96%	67%	IPR000306, IPR003903, IPR011011, IPR013083, IPR017455
FQ867273	Rab7	627	similar to putative Rab7 isoform 1	*Nasonia vitripennis*	XP_001607917	1E-97	99%	83%	IPR001806, IPR002041, IPR003577, IPR003578, IPR003579, IPR005225, IPR013753, IPR020851
FQ878926, FQ877422, FQ883262, FQ879701	FK506BP	1380	hypothetical protein TcasGA2_TC008405	*Tribolium castaneum*	EFA02684	9E-176	99%	67%	IPR001179, IPR011990, IPR013026, IPR013105, IPR019734, IPR023566,
FQ866673	MEGwB	390	similar to allergen Aca s 13 (FABP-like protein)	*Tribolium castaneum*	XP_969762	0.00003	82%	32%	IPR011038, IPR012674
